# Comparison of Two Intracanal Irrigants’ Effect on Flare-Up in Necrotic Teeth

**Published:** 2007-01-20

**Authors:** Mina Zarei, Maryam Bidar

**Affiliations:** 1 *Department of Endodontics, Dental School, Mashad University of Medical Sciences, Mashad, Iran*

**Keywords:** Chlorhexidine Gluconate, Flare-Up, Sodium Hypochlorite

## Abstract

**INTRODUCTION:** The aim of this study was to compare the efficacy of two irrigants on decreasing the pain and swelling at different times after treatment of necrotic pulp.

**MATERIALS AND METHODS:** Fifty patients with single canal tooth and necrotic pulp were selected and divided into two groups, twenty-five in each. Rotary files were used for preparing the canals and 0.2% chlorhexidine gluconate and 2.5% sodium hypochlorite were used for irrigation of canals. Then canals were filled by lateral condensation technique. A questionnaire was given to patients asking for the level of their pain and swelling. The patients were followed for 48h. Visual Analogue Scale (VAS) was used for determination of pain degree. The scale with 4 levels was used for measurement of the intensity of swelling. The data were statistically analyzed using Mann-Witney and Kruskal-Wallis tests.

**RESULTS:** The research showed no significant difference between irrigant solutions in decreasing the amount of pain and swelling after endodontic treatments. No significant relationship was detected between the incidence of pain with swelling, age, and sex. Flare-up in maxilla was more than mandible.

**CONCLUSION:** According to results of this *in vivo* study it was concluded that efficacies of 0.2% chlorhexidine gluconate and 2.5% NaOCl are the same.

## INTRODUCTION

"Flare-up" can cause problem for patients and dentists the both (1). Different studies had shown various results about causative factors of flare-up. Factors such as necrotic pulp, preradicular pain, age, race, gender, tooth position and materials have been evaluated for this reason (2-8). Sim reported a significantly higher incidence of flare-up in necrotic teeth than vital teeth (4), but others showed contraversial results (9).

Microbial, chemical or mechanical injuries to pulp or periradicular tissue and specially microrganisms and their by products are the most important reasons of pulpal or periapical inflammation (10-12). Removing the micro-rganisms has a direct effect on the success rate of endodontic treatment (13). Pushing the infectious debris to periapical region and inadequate chemical and mechanical cleaning are the reasons for inflammation of periapical region (10). So a complete removal of necrotic and infectious debries from root canal system is very important.

Many bacterias, as observed in an infectious canal, were removed from canal during root canal treatment, but despite of adequate cleaning, many remain in anatomical complexities of root canals (14).

In fact, only 50% of bacteria were removed from canals (12). Therefore, the materials should be used during the preparing of canal so that it can remove more debris, necrotic pulp and microrganism. Using irrigant solutions in root canal therapy has an important role in successful endodontic treatments (14). Sodium hypochlorite (SH) is one of the most effective irrigants with antibacterial properties. It can irrigate canal and dissolves its vital and necrotic tissues (15). SH has particular toxic affects on periapical tissue in high concentration, and causes acute inflammation (12,13,16,17). On the other hand, a bad odour, caustic effect of material and other undesirable properities like corosion and color change of divices, made researchers find another irrigant (18). In addition, sodium hypochlorite with adequate antibacterial effects has short effect and no durability (15-16). Harrison *et al.* in a clinical study on toxic properties of 5.25% SH, observed no significant difference in postoperative pain in cases which were irrigated with either SH or normal salin. They declared that toxicity of 5.25% SH is not more than normal saline (19).

Nowadays, chlorohexidine gluconate (CG) is considered because of antibacterial effect, durablility and it's non toxic property as irrigant (11). White *et al.* studied on durability of antibacterial effects of CG in 0.2% and 0.12% concentration, and they found that 0.2% solution has more considerable durablity. Antibacterial activity of 0.2% CG remained for 72h after cleaning the canal (20).

Others compared antibacterial effect of two irrigants, 0.2% CG and 5.25% SH with salin and showed that their cultured possitives were less than salin (21). Menezes *et al.* compared antibacterial effect of 2.5% SH and 0.2% CG with six another irrigants. They found that CG was more effective on *E. Faecalis* (22).

The aim of this *in vivo *study was to compare the efficacy of two irrigants on the pain and swelling in different periods after necrotic pulp treatment.

## MATERIALS AND METHODS

In this study 50 teeth were cured. Selected patients did not have any systemic disease, analgesic or antibiotic medication since two weeks before the study. All teeth were necrotic with no sign or symptom.

The patients were divided into two groups, and selected teeth were anesthetized using Persocaine (Lidocaine HCl+ Epinephrine 1/80000). 2.5% SH and 0.2% CG were used for appropriate groups. All teeth were isolated after removing of carries and access cavity preparation. Using K-file, working length was established by periapical radiography at 0.5-1mm of apical foramen. Rotary files (Easy-Race) were used for canal preparation. Root canals were prepared by crown-down technique. After each file removal, 1.5-2 ml irrigant was used for each group. Canals were dried with praper points. Root canals were filled with gutta-percha and AH26 sealer by lateral condensation technique. The tooth were then temporary filled with cavit.

**Table 1:** Severity of swelling after 48h in jaws

**Swelling**	**No**	**Mild**	**Moderate**		**Severe**
**Maxilla**	1(3.2%)^a^	1(3.2%)	4(12.9%)	25(80.6%)	
**Mandible**	0	0	0		19(100%)

a Number (Percent);

Results of man-witnney test:P Value= 0.04

Two VAS forms were given to the patients for measuring of the pain and swelling. The VAS pain form was scaled from 0-9 and patients marked the appropriate level of the pain in specified times. Data were collected and classified in 4 groups: no pain, mild, moderate, and severe. The swelling was classified in 4 scales: no swelling, mild, moderate, and severe. The patients were followed-up for 48h. Data were analyzed by Mann-Whitney an Kruskal-Wallis tests.

## RESULTS

**Figure 1:**
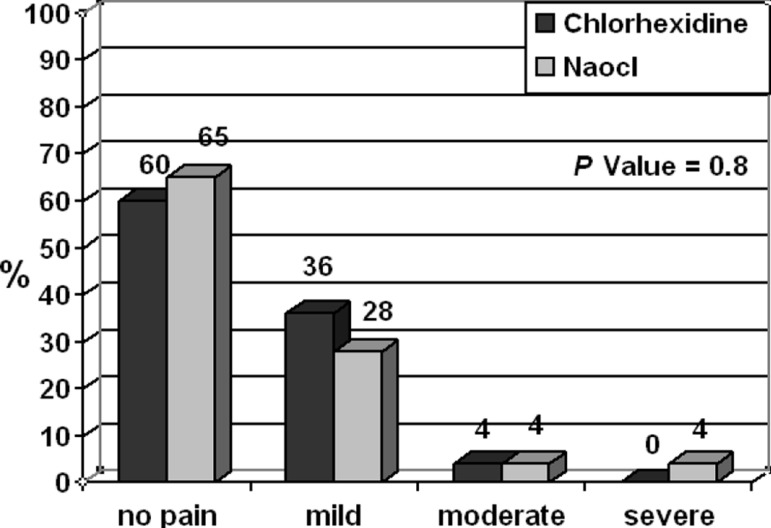
Value of the severity of pain

**Figure 2:**
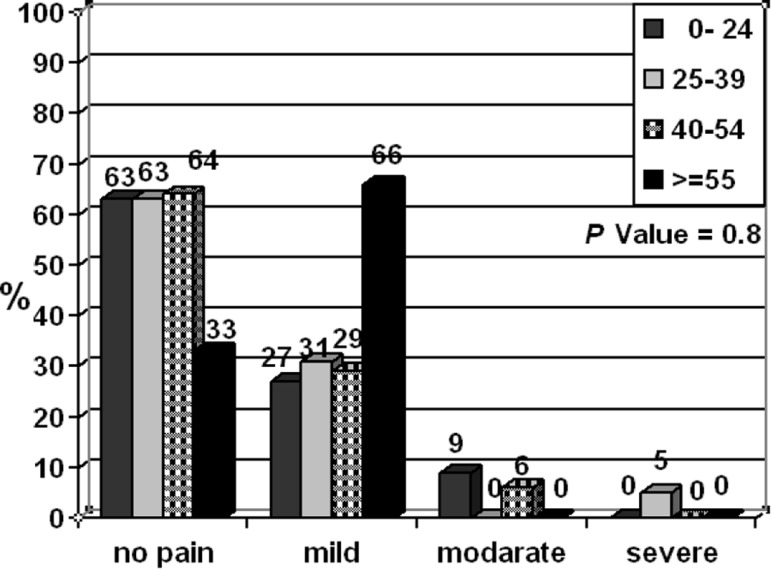
The pain in different ages

In this study, no significant difference was found for the incidence of pain in two groups for each period of time. According to the Mann-Witney test, the incidence of pain in two groups were analougous (Figure 1). The camparison of two groups in times periods of 6,12,18,24, and 48h showed no statistically significant difference between severity of pain and swelling after treatment. Relationship between pain severity and age is demonstrated in Figure 2.

According to Table 1 no significant difference was detected in the incidence of swelling in jaws 24h after treatment, while more swelling was observed in maxilla after 48h (P<0.05).

## DISCUSSION

Acute pain and swelling after treatment was considered as flare up (1). Although it is proved that flare-up does not affect the result of the endodontic treatment, but it has inappropriate effect on relationship between patient and dentist (23-25). A positive correlation between Flare-up with single and multiple appointment, retreatment cases, periradicular pain, and radiolucent lesions has been reported, while no correlation between post obturation flare-up and the pulp status was detected (3).

However, Sim reported higher incidence of flare-up in necrotic teeth (4). Because of these contraversies, we selected the necrotic teeth with no sign or symptom.

There are many studies about antibacterial properties of irrigant solutions (24,26,27). SH is usually considered because of its antibacterial property and adequate dissolving of pulp tissue, but it is toxic for periapical tissues. CG is a durable antibacterial irrigant (22,28,29). In this research, the effect of two above irrigants on decreasing of the pain and swelling on necrotic teeth after treatment is compared. Many researchers have already studied about antibacterial effects of these irrigants, but their effects on flare-up has not been studied yet.

Crown-down technique with rotary files were used, in this study, for better cleaning of canal. Marshal *et al.* showed that this technique affected the prevention of problems following treatment and inadequate irrigation (30).

Researchers found no significant difference between antibacterial effect of CG and 2.5% SH (17). Others didn't find any significant difference on positive cultured samples between 5.25% SH and 0.2% CG (21).

In our study in SH group, the severe pain was recorded in all intervals, while in CG group the strain wasn't observed after treatment except one case in the first 12h after treatment. The amount of strain pain in SH group was more than the CG group, but it is not statistically significant.

The amount of severe swelling was observed in SH group in two time intervals, but in CH group no severe pain was recorded. No relashonship was obsereved between the incidence of pain with age and sex. Yeh *et al.* found no relashionship between value of flare-up with age and sex (31). Zoulo and Imura also found no relashionship between the incidence of flare-up with age and sex (3).

The incidence of the pain has been studied in maxilla and mandible, but in spite of the results of Torabinejad study (32), the severity of pain in maxilla was more than mandible. The recorded amount of swelling in maxilla was also more than mandible in 48h after treatment.

## CONCLUSION

In this research, SH and CG had equal effect on severety of pain and swelling after treatment. So, 0.2% CG can be recommended as an acceptable irrigant for endodontic treatments.
